# Enriching WPCs and NFPCs with Carbon Nanotubes and Graphene

**DOI:** 10.3390/polym14040745

**Published:** 2022-02-15

**Authors:** Damian Łukawski, Patrycja Hochmańska-Kaniewska, Dominika Janiszewska, Grzegorz Wróblewski, Jeff Patmore, Agnieszka Lekawa-Raus

**Affiliations:** 1 Institute of Physics, Faculty of Materials Engineering and Technical Physics, Poznan University of Technology, Piotrowo 3, 61-139 Poznan, Poland; damian.lukawski@put.poznan.pl; 2 Wood Technology Centre, Łukasiewicz Research Network—Poznań Institute of Technology, ul. Winiarska 1, 60-654 Poznan, Poland; patrycja.hochmanska@pit.lukasiewicz.gov.pl (P.H.-K.); dominika.janiszewska@pit.lukasiewicz.gov.pl (D.J.); 3 Faculty of Mechatronics, Warsaw University of Technology, ul. św. Andrzeja Boboli 8, 02-525 Warszawa, Poland; grzegorz.wroblewski1@pw.edu.pl; 4 Pembroke College, University of Cambridge, Trumpington St., Cambridge CB2 1RF, UK; jjp43@cam.ac.uk

**Keywords:** wood–plastic composites, natural fibre–plastic composites, carbon nanotubes, MWCNTs, graphene, graphene oxide, graphene nanoplatelets, hybrid composites

## Abstract

Carbon nanotubes (CNTs) and graphene, with their unique mechanical, electrical, thermal, optical, and wettability properties, are very effective fillers for many types of composites. Recently, a number of studies have shown that CNTs and graphene may be integrated into wood–plastic composites (WPCs) and natural-fibre-reinforced polymer composites (NFPCs) to improve the existing performance of the WPCs/NFPCs as well as enabling their use in completely new areas of engineering. The following review analyses the results of the studies presented to date, from which it can be seen that that inclusion of CNTs/graphene may indeed improve the mechanical properties of the WPCs/NFPCs, while increasing their thermal conductivity, making them electroconductive, more photostable, less sensitive to water absorption, less flammable, and more thermally stable. This study indicates that the composition and methods of manufacturing of hybrid WPCs/NFPCs vary significantly between the samples, with a consequent impact on the level of improvement of specific properties. This review also shows that the incorporation of CNTs/graphene may enable new applications of WPCs/NFPCs, such as solar thermal energy storage devices, electromagnetic shielding, antistatic packaging, sensors, and heaters. Finally, this paper recognises key challenges in the study area, and proposes future work.

## 1. Introduction

Wood–plastic composites (WPCs) entered the market in the 1990s; they are usually composed of up to 50% *w*/*w* of wood flour, thermoplastic polymers (polypropylene, polyethylene, polyvinyl chloride etc.), and small amounts of additives such as dyes or coupling agents [1]. WPCs are most often used in construction and flooring, replacing lumber, wood-based boards (WBBs), ceramic tiles, or metals. WPCs can be designed for specific performance requirements, taking into account the advantages of the natural strength of the wood and the hydrophobic characteristics of the plastic component. The possibility of use of wood waste fillers as well as waste and recycled plastics reduces the environmental impact of the material, and also improves the circular economy [2,3,4]. Other advantages of WPCs are their ease of maintenance, improved durability, and substantial service lifetime [5]. The large weight share of polymers enables the shaping of WPC elements by moulding. Moulding allows the formation of complex shapes that would be very costly or even impossible to produce with pure lumber, wood-based boards (WBBs), or stone/ceramics. Furthermore, ready-made WPCs may be further shaped using the same tools as conventional lumber.

However, WPCs are also not free from disadvantages. The large weight share of the polymer matrix also predetermines the mechanical properties of the WPCs, which are close to those of their base polymers; therefore, they are poorer than (for example) raw wood. Another negative property that has been observed in WPCs is their susceptibility to environmental degradation. This type of degradation can occur as a result of various factors, including temperature, air quality, moisture (the polymer matrix does not fully exclude water interaction), microorganisms, light, high-energy radiation, chemical agents, and mechanical stress [6,7]. These factors can decrease the aesthetic quality (discoloration) and mechanical strength of WPCs [1,8,9]. Moreover, evidence has been seen of the presence of fungal attack on WPC boards that are exposed to natural weathering [10,11].

Even taking account of these issues, the current wide use of WPCs, coupled with their composite nature, favours the development of research in this area. Very often, the studies are extended to the use of other cellulose-rich materials (e.g., milled straw, plant shells, or bamboo) as fillers [12]. The latter composites are referred to as natural fibre–polymer composites (sometimes WPCs are classified as subgroup of NFPCs). NFPCs are not yet widely applied, as up to this point their performance has often been found to be less than satisfactory [12]. However, new studies in this area show very encouraging results [13,14,15,16,17,18]. Other studies focus mainly on the improvement of the performance of the WPCs, utilisation of new types of matrices, new manufacturing techniques, and an increase in the range of applications [19,20,21,22,23]. One of the notable interesting areas of study is in the enrichment of the WPC and NFPC composites with carbon nanomaterials, such as carbon nanotubes (CNTs) and graphene. 

**Figure 1:**
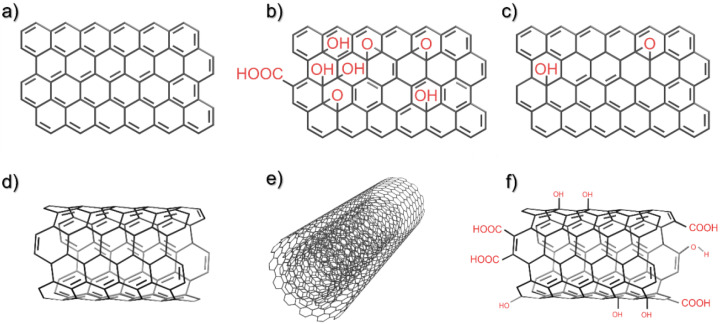
Structure of (**a**) graphene, (**b**) graphene oxide, (**c**) reduced graphene oxide, (**d**) single-walled carbon nanotube (**e**) multiwalled carbon nanotube, and (**f**) functionalised carbon nanotube. Annotation indicates where the structure is not pure carbon.

Both carbon nanotubes and graphene are nano-allotropes of pure carbon [24,25]. The structure of both is based on a planar hexagonal lattice of sp^2^-bonded carbon atoms (Figure 1a), i.e., every carbon atom forms a strong covalent bond with three neighbouring carbon atoms, and a much weaker pi bond is formed by the atoms laterally by their fourth available electrons distributed in *p_z_* orbitals perpendicular to the hexagonal plane. This atomic structure imparts both CNTs and graphene with extremely high mechanical strength and stiffness, as well as superior electrical and thermal conductivity [26]. Additionally, both of these nanomaterials are lightweight [27], superhydrophobic [28,29], chemically resistant [30], and environmentally friendly, as they may be synthesised from greenhouse gases such as methane or CO_2_ [31,32]. 

Graphene is a 2D structure that, in theory, is one atomic layer thick (Figure 1a). In practice, the name graphene often refers to the whole family of two-dimensional carbon nanomaterials. The material to mention first in the context of WPCs is graphene nanoplatelets (GNPs); these are multilayered pure graphene structures often produced by the exfoliation of graphite, or via the chemical vapour deposition (CVD) process. Although the properties of GNPs may not be superior to those of single-layer graphene, they are sufficient to improve the properties of many polymer composites [33]. GNPs are also quite inexpensive, easy to handle, and are produced and sold in large quantities. A further material is graphene oxide (GO) (Figure 1b), which is a disordered single- or multilayered graphene functionalised with epoxy bridges, hydroxyl groups, and carboxyl groups [34]. GO is electrically insulating, and has a much poorer mechanical performance than graphene/GNPs, but it is also a chemically active and hydrophilic structure; this enables dispersion in water and a uniform distribution in many polymers [35]. Finally, reduced graphene oxide (rGO) is a material formed by the chemical reduction of GO. This process partially restores the electrical and mechanical properties of the graphene via the removal of most of the functional groups and the recovery of the sp^2^ carbon–carbon bonds (Figure 1c) [36]. Often, the reduction process is performed after the composite manufacturing process, so as to benefit from the chemical reactivity of GO during manufacture and the electrical and mechanical properties of graphene in the final product. 

Carbon nanotubes are one-dimensional structures of seamlessly rolled-up graphene sheets; they may be referred to as single-walled carbon nanotubes (SWCNTs) if made of a single layer of graphene (Figure 1d), or multiwalled carbon nanotubes (MWCNTs) if two or more SWCNTs are encapsulated within one another (Figure 1e). SWCNTs are often characterised as having better properties than MWCNTs, but the MWCNTs can be produced inexpensively and on a large scale [31]. The CNTs may also be produced in a functionalised form to facilitate the dispersion of these structures in polymer matrices (Figure 1f) [37]. As 1D structures, the CNTs have nanosized diameters, and are most often nano- or micrometre lengths. In composites, CNTs form percolation networks more easily than graphene, but in some processes it is more difficult to obtain their high loading fractions [38,39].

Both graphene and CNTs have been widely used in composites [40]; they have been tested as fillers in many types of matrices, including polymers [39,41], metals [42], and ceramics [43]. Polymer composites were actually the first major area in which CNTs and graphene found commercial applications. Both nanomaterials have been used to enhance mechanical properties [44], improve thermal stability [45], decrease flammability [46,47], introduce electrical and thermal conductivity [39], and much more [48]. The applications of these composites include strong and lightweight construction materials [43], electromagnetic shielding [49], antistatic surfaces [50], sensors [51], components such as supercapacitors, etc. [52]. It may therefore be expected that a combination of WPCs and other NFPCs with CNTs and/or graphene may produce materials with very interesting performance parameters, suitable for completely new applications. 

The following study focuses on the literature presented to date in the area of WPC/NFPCs enriched with CNMs. The study shows the potential ways in which CNMs can improve the performance of NFPCs, and analyses the factors that may influence the properties of the NFPC–CNM hybrid composites. The properties studied to date are covered in Section 2.1, Section 2.2, Section 2.3, Section 2.4, Section 2.5 and Section 2.6 (2.1 Mechanical Properties; 2.2 Electrical and Thermal Properties; 2.3 Photostability; 2.4 Water Absorption and Swelling Thickness; 2.5 Thermal Stability and Flammability; 2.6 Foaming Efficiency). Finally, the study provides examples of new applications that may be targeted as a result of the addition of this new filler. 

## 2. Improvement of WPC/NFPC Performance by the Addition of Carbon Nanomaterials

The existing literature indicates that enriching WPCs and NFPCs with carbon nanomaterials may be highly beneficial to the performance of the WPCs/NFPCs. Expanding on this, in general, it has been shown that the addition of graphene or carbon nanotubes may result in an improvement of mechanical properties [53,54,55,56], an increase in electrical and thermal conductivity [57,58,59,60], a decrease in water absorption, and an improvement of thermal stability [61,62] and/or fire retardancy [60]. All of these tested properties are listed in Table 1. 

**Figure 2:**
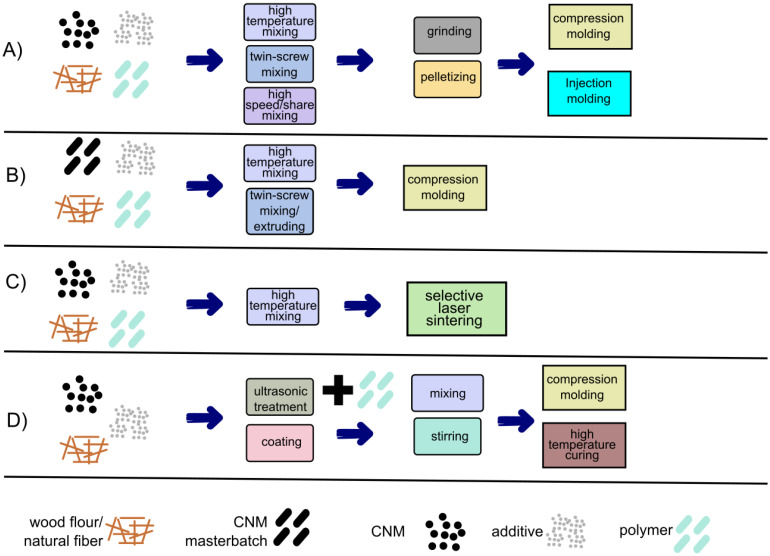
The scheme of various processes used when producing CNM-reinforced WPC. (**A**) compression/injection moulding with CNM powder, (**B**) compression moulding with CNM masterbatch, (**C**) selective laser sintering, (**D**) compression moulding/high temperature curing with wood flour coated with CNM suspension.

Table 1 also lists the composition of the WPCs/NFPCs, the type of CNM used, and the method of production (these production methods are explained graphically in Figure 2). It can be seen that the studied WPCs/NFPCs differed significantly in composition. The composites were based on thermoplastic [53,54,57] and thermoset matrices [70]; they used varying weight percentages of wood or cellulosic particles and different types of wood and cellulosic components as fillers [53,54,55,70]. Most of the composites included additives to facilitate the formation of high-performance materials [53,54,63]. Different composite manufacturing techniques were also proposed [53,54,55,70] (Table 1, Figure 2). The CNMs used in the studies shown in Table 1 included graphene nanoplatelets [70], functionalised graphenes [53], graphene oxides [61], reduced graphene oxides [58], carbon nanotubes (multiwalled) [54,55,68], and functionalised carbon nanotubes [77]. No less important is variety of techniques used for the incorporation of the CNMs.

This large variety of materials and manufacturing techniques indicates that the skilled incorporation of CNMs into WPCs/NFPCs may improve the properties of almost any of these composites. However, obtaining the optimal composition with its associated manufacturing method (one that will enable specific properties and levels of improvement to be achieved) is at present still challenging and difficult to foresee. 

It has often been reported in the literature that the properties of WPCs/NFPCs depend strongly on their composition and manufacturing techniques [78,79,80,81,82,83,84]. In every study shown in Table 1, different base/reference materials are considered; therefore, we may expect variations in the properties observed. Similarly, the literature on CNM composites shows that the properties of the composites are dependent on the type, quality, and weight percentage of the CNMs, along with their compatibility with the matrix and the composite manufacturing method used [38,85,86,87]. As shown in Table 1, these factors are also changed significantly in the WPC/NFPC–CNM hybrids. 

An in-depth comparative analysis of all of the papers listed in Table 1 enabled us to provide some general conclusions with regard to the design of the WPC/NFPC–CNM hybrids with specific properties, as well as indicating the areas for further research. 

### 2.1. Mechanical Properties

To date, most publications in the area of WPC/FNPC–CNM hybrids have concentrated on their mechanical properties, either focusing the whole study on mechanical properties or performing mechanical tests alongside tests of other properties. Studies of mechanical performance mainly include tensile and flexural/bending tests; properties such as impact strength, storage and loss modulus, and scratch hardness also appear. In most cases, the improvements measured were in the range of single-digit percentages to tens of percent; however, occasionally they achieved improvements of hundreds of percent. 

An example is a study by Sheshmani et al. [63], who reported enriching a WPC with graphene nanoplatelets. The WPCs were based on poplar flour, and used polypropylene and maleic anhydride-grafted polypropylene (MAPP) as coupling agents. In every composite, the poplar flour constituted 20 wt.%, MAPP 3 wt.%, and graphene 0.2, 0.4, 0.6, 0.8, 1, 2, 4, or 5 wt.%. The samples were prepared by mixing at an elevated temperature, grinding the cooled blends and, finally, compression moulding. It was found that the best improvement of mechanical properties was obtained at 0.8 wt.% GNP. The tensile strength and modulus increased by almost 20% with reference to a WPC without graphene. The flexural strength improved by 54%, while the flexural modulus increased by 41%. The elongation at break remained at the same level; however, the impact strength increased by 115%. 

For comparison, Ye et al. [61] manufactured samples with the addition of graphene oxide (GO). The base WPC was fabricated from polypropylene (PP), branched polyethylenimine (PEI), and recycled poplar powders. GO, PEI, and poplar powders were combined into PEI–wood–GO particles in a multistage sonication-assisted process. The PEI–wood–GO particles were then mixed with PP, melted, and compounded in torque rheometers, with a ratio of 40 wt.% PEI–wood–GO and 60 wt.% PP. The weight fractions of GO in the final WPC were set at 0.1, 0.2, 0.3, and 0.4%. The presence of GO improved the mechanical properties of the composites. The tensile strength increased by 57.86%, the elastic modulus by 48.92%,the elongation at break by 70.17%, the flexural strength by 26.31%, and the flexural modulus by 75.21%; the largest flexural force was increased by 21.89% with regard to the reference PEI–WPC. 

Another example was published by Nourbakhsh et al. [54], who formed a set of samples using either poplar fibres or bagasse stalk with a polypropylene/MAPP coupling agent and MWCNTs. The ingredients were compounded by the use of a co-rotating twin-screw extruder, and then granulated and injection moulded. All samples included 40 wt.% percent natural fibres. The other 60 wt.% was divided between PP and MAPP (3 wt.%)—a reference sample—or PP, MAPP (3 wt.%), and MWCNTs (1.5, 2.5, or 3.5 wt.%). In most cases, the best results were obtained from a sample containing 2.5 wt.% MWCNTs. From these samples, rough estimations were produced based on the presented figures, and showed that for the poplar sample cases the tensile strength increased by almost 30% with regard to the reference sample (40 wt.% fibre, 57 wt.% PP, and 3 wt.% MAPP) with the addition of 2.5 wt.% MWCNTs. The tensile modulus increased by over 20%, while the flexural strength and modulus were 7% and 9% higher, respectively. The impact strength appeared to be the highest for 1.5 wt.% content of MWCNTs, and increased by approximately 13% with regard to the reference sample. The mechanical properties of the bagasse samples were in most cases poorer than the poplar samples; however, all showed an increase in strength and stiffness upon the addition of MWCNTs. 

Kushwaha et al. [77] presented WPC samples that differed from previously presented samples by virtue of the fact that they were prepared from bamboo strip mats rather than microparticles of wood/natural fibres. The bamboo mats were treated with an alkali prior to compounding with epoxy resin (CY-230), hardener (HY-951), and plasma-treated carbon nanotubes. The composites were prepared using a hand lay-up technique. The addition of plasma-treated CNTs produced an improvement in tensile strength (6.67%), tensile modulus (2.7%), flexural strength (5.8%), flexural modulus (31%), and impact strength (84.5%). 

**Figure 3:**
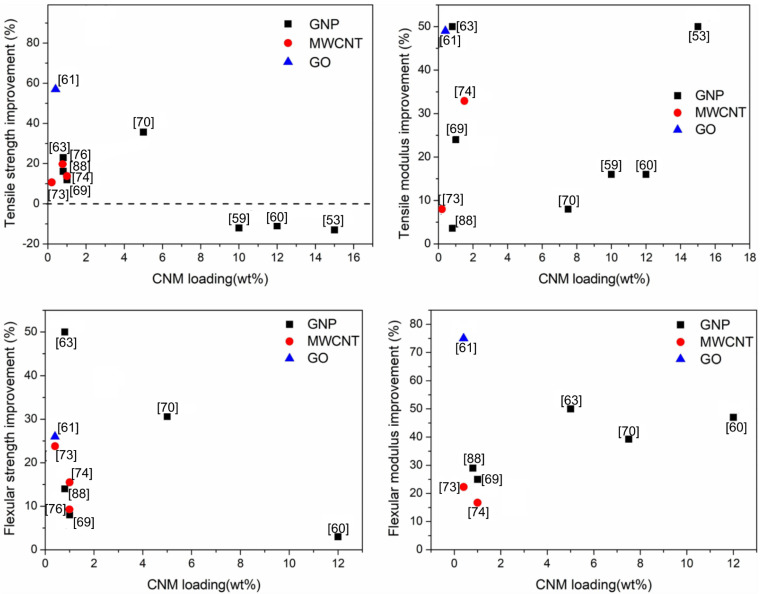
Mechanical properties of CNM-enriched WPCs, based on recent papers. The presented values present the best performing sample in each work.

The studies described above showed that an improvement in the mechanical properties may be obtained through the addition of different nanoforms of carbon with various natural fillers. However, the exact percentage improvement of specific properties differs from sample to sample (Figure 3).

An important conclusion of the work of Nourbakhsh et al. [54], who tested samples with different natural fillers, is that the properties of the base WPC may significantly affect the properties of the CNM-enriched composite. For example, the tensile strength of the base WPC with poplar wood fibres amounted to approximately 33 MPa, and for the bagasse stalk fibres approximately 31 MPa—a relatively small difference. Analogous composites enriched with 2.5 wt.% MWCNTs reached approximately 63 MPa and 44 MPa for poplar- and bagasse-based samples, respectively. In the case of the tensile modulus, the initial values for the reference WPC samples showed a much larger difference (2008 MPa for the poplar sample and 3100 MPa for the bagasse stalk sample), while the respective values for the 2.5 wt.% MWCNT-enriched samples changed by almost the same values—795 MPa and 768 MPa for poplar and bagasse samples, respectively. 

The study of Kumar et al. [70] highlighted the fact that that the amount of natural filler is of importance. The samples were prepared via the mixing of LY-556 epoxy resin and HY-951 hardener (ratio of 10:1) with 0.5 wt.% GNP and 2.5, 5, 7.5, and 10 wt.% mixed wood particles. The mixture was then sonicated, degassed in a vacuum, poured into a mould, and polymerised under ambient conditions. The best mechanical performance was obtained for samples with 5 wt.% wood particles. For these samples, an increase in the tensile strength of 35.55% was observed, compared to samples without wood particles. The flexural strength increased by 30.64%, hardness by 22.98%, impact strength by 41.67%, conductivity by 16.05%, fracture toughness by 26.71%, and fracture energy by 74.38%. Unfortunately, as all of the samples contained 0.5 wt.% GNP, it was not shown how the amount of wood particles cooperates with the effect of the GNPs. It would be very useful to perform the same studies with various amounts of GNPs and without the CNM filler. 

Ge et al. [64] tried to see how the overall performance of the composites is dependent on the ratio of wood particles to polymer matrix, as well as on the carbon nanomaterial type. The authors prepared a set of samples using 30, 40, or 50 wt.% decayed wood particles (*Pinus massoniana Lamb*), with 2% of the total mass of the chosen nanomaterial being either carbon nanotubes, graphene, activated carbon, or bamboo charcoal; 3 wt.% chitosan was also added with the aim of improving the interface properties of the composite, and polyvinyl chloride (PVC) was used as a matrix material. For the samples without any carbon filler, the highest values of damage load, bending strength, and elastic modulus were recorded for 40 wt.% wood particles; however, the tensile strength for this composition was the lowest. The addition of various carbon materials gave rather scattered results, both improving the absolute obtained values and decreasing them, as well as producing better results for 30 wt.% or 50 wt.% wood flour. This is an important result, and indicates that the natural and CNM fillers may influence the effectiveness of one another. The authors proposed that the optimal composition should be 40 wt.% wood particles, 60 wt.% PVC, with 2 wt.% CNTs and 3 wt.% chitosan. Indeed, this sample achieved the best tensile strength of all of the samples, and had better damage load and bending strength than any WPC sample without carbon-based reinforcement and sufficiently good elastic modulus. 

Furthermore, Kaymakci et al. [67] showed that the effectiveness of CNMs in the improvement of WPC properties may be dependent on the presence of compatibilizers. Kaymakci et al. tested the surface roughness, wettability, and scratch properties of MWCNT-enriched WPCs. The samples were composed of 50/50 *w*/*w* pine wood flour and polypropylene. The MWCNTs constituted either 0 or 1, 3, or 5 wt.% of the composite. There were also two versions of the composites with MAPP (3 wt.%) and without MAPP. It was found that the increasing content of MWCNTs significantly decreases the surface roughness of the samples. The sole presence of MAPP also increases the effect; however, its impact is less than that of the nanotubes. The addition of both fillers produces almost the same surface roughness as in the case of MWCNTs alone (5 wt.%). Both MAPP and MWCNTs decreased the wettability of the samples. The most beneficial change was in the presence of both MAPP and MWCNTs, as a superposition of both effects was observed; however, the effectiveness of MWCNTs slightly dropped when MAPP was present. The scratch hardness was also improved by both MWCNTs and MAPP; however, although it clearly increased with the content of MWCNTs, the increase induced by 5 wt.% CNTs was less than that of 3 wt.% MAPP. Again, the best results were obtained when the effects of both MAPP and MWCNTs were superimposed. 

The study of Nabinejad et al. [73] showed that the method of manufacture of the hybrid WPC is very important. The samples were composed of oil palm shell powder (15 phr), an unsaturated polyester (UP) matrix, and various amounts of MWCNTs (0.2, 0.4, 0.6, and 0.8 phr). The preparation method was performed with and without the aid of a solvent. The tested solvents included styrene, ethanol, methanol, and acetone. It was found that the most effective curing of the UP resin took place in the presence of styrene. Styrene samples achieved the highest tensile and flexural properties. 

Wang et al. [75] and Gouda et al. [88] claimed that the properties of their NFPCs improved thanks to the chemical modification of their natural bamboo fillers. Wang et al. [75] grafted GO into bamboo fibres treated with 1 wt.% NaOH. The mixture was then blended with melted PP and injection moulded. The tensile and flexural strength tests showed that the alkali treatment of the bamboo fillers improved the properties of the base WPC, and that the addition of GO further improved these parameters. However, no reference studies showing the performance of WPC with untreated bamboo fillers and GO were provided. Therefore, it is difficult to judge the influence of the alkali treatment of the bamboo filler on the GO’s effectiveness. A similar conclusion may be drawn with regard to the study of Gouda et al. [88].

Most of the presented studies focused on the role of carbon nanomaterials and the methods of achieving the best performance with their use. As shown in the study of Ge et al. [64], the properties of hybrid WPCs are dependent on the type of carbon nanomaterial. However, it is important to mention that not only the type of carbon nanomaterial (CNTs, graphene, graphene oxide, etc.), but also the internal properties of the given CNM—such as the number of layers/walls, length/diameter, defectiveness, etc.—may influence the properties of the composite [66]. The performance of a specific material is strongly related to the process of synthesis. At present there are few standards with regard to the manufacturing of carbon nanomaterials; therefore, similar materials made by various manufacturers may produce composites of different properties. This issue was partly covered by Al-Maqdasi et al. [53], who prepared WPC samples by the mixing of sawdust of spruce and pine wood with high-density polyethylene (HDPE), a compatibilizer—maleic anhydride-grafted high-density polyethylene (MAPE)—and two types of masterbatches of graphene platelets oxidised at the edges in HDPE. It was found that, overall, better results are obtained from a graphene M2 masterbatch with platelets of 6–10 layers and a flake size of 38 µm than for an M1 graphene masterbatch with average thickness of 20 nm and flake size of 50 µm. These results show that for a sample containing 40 wt.% wood flour, the addition of 15 wt.% M2 graphene caused the stiffness and yield strength to increase by 50% and 14%, respectively. In the case of this study, GNPs also improved the flexural modulus; however, they had almost no influence on flexural and impact strength [89]. 

Several of the above papers note that the properties of the composites improved with the increased loading of CNMs. However, increasing the loading above a specific point led to a renewed reduction in the performance. In the case of Sheshmani et al. [63], the best mechanical performance was obtained with 0.8–1wt.% graphene nanoplatelets. A higher loading of GNPs did not produce a sufficient improvement in the properties. A microscopic study showed that 3–5 wt.% GNPs resulted in agglomeration of the nanomaterial and, therefore, a poorer performance of the composite. Analogous results were obtained by Nourbakhsh et al. [54], who found that the best mechanical properties of the samples were obtained for 2.5 wt.% MWCNT loading; a 3.5 wt.% loading of MWCNTs resulted in their agglomeration and, therefore, a much poorer stress transfer. Nabinejad et al. [73] showed that in the case of thermoset polymers an increase in the content of CNTs caused a very high increase in viscosity, and resulted in a very high void content; this also caused a reduction in the mechanical properties at high weight percentages of the CNTs. 

To deal with this issue, Ye et al. [61] first combined the wood powders with GO using PEI in a multistage sonication-assisted process. The PEI–wood–GO particles were then combined with a PP matrix. It is rather difficult to judge how effective this process would be for higher loading fractions of GO, as the highest GO loading produced was 0.4 wt.%. 

Zhang et al. [55] prepared WPC samples via the selective laser sintering of polyether sulfone and pine wood powders (mass fraction ratio of 6:1). To improve the load transfer, the samples were subjected to additional microwave treatment. The authors explained that this treatment remelted the polymer matrix around the MWCNTs, improving the interface of the CNM and the matrix. In comparison, the addition of 0.1 wt.% MWCNTs to the basic process resulted in an approximately 90% increase in bending strength. Further microwave treatment of the samples increased the bending strength by another 4.2–64.2% with regard to the base laser-sintered CNT-enriched WPC. Again, in this case, the loading of the CNM was low. 

It seems that the most effective method of dealing with the agglomeration of CNMs and poor load transfer was found by Al-Maqdasi et al. [53], who used masterbatches of graphene nanoplatelets oxidised at the edges. The oxidised graphene nanoplatelets were mixed with high-density polyethylene (HDPE), which was also used as a matrix for the prepared WPC. The loading fractions obtained by the authors were as high as 15 wt.%, and the best mechanical properties were obtained for these loadings, indicating that no agglomeration took place. 

The last issue to note with regard to loading-dependent changes in mechanical performance is the fact that the maximum improvement of different properties may take place for slightly different percentages of carbon fillers. This phenomenon may be described based on the studies of Yaghoobi et al. [72,74]. The tested composites were based on PP, MAPP, kenaf fibres, and MWCNTs. Premixed PP and MAPP powders were melted and blended with MWCNTs and kenaf fibres. Cooled blends were then hot-press-moulded. The samples contained 30 wt.% kenaf fibres, 5 wt.% MAPP, and 0–2 wt.% MWCNTs. It was found that the addition of MWCNTs increased both the storage and loss moduli. The highest increases in tensile strength, flexural strength, and notched impact strength were observed for 1 wt.% MWCNTs, and amounted to 13.8%, 15.6%, and 11.4%, respectively. Meanwhile, the tensile and flexural moduli improved the most for 1.5 wt.% CNTs—by 18.9% and 17%, respectively. The highest increases in the storage and loss moduli were also observed for 1.5 wt.% CNTs. At 25 °C, the value of the storage modulus increased from 1995 MPa to 2623 MPa, while for the loss modulus this was from 140 MPa to 180 MPa. The above phenomenon may be explained by the different structural changes taking place during the varying mechanical tests. It should be noted that not all of them will be similarly affected by agglomeration or voids. 

### 2.2. Electrical and Thermal Conductivity

It is important to highlight that in some cases agglomeration may not be an issue. It has been shown by several authors that WPCs may become electroconductive upon the addition of CNMs. The electrical conductivity of WPCs increases with the loading fraction of CNMs, and is not deteriorated by their poor distribution in the matrix. Zhang et al. [57] fabricated WPC samples from poplar wood fibres, polyethylene, and anhydride-grafted polyethylene (MAPE) as a coupling agent and lubricant, as well as either MWCNTs, flake graphite, or carbon black. The samples were prepared in a twin-screw one-step extruder system. The weight ratio of wood fibres to polyethylene was approximately 6:4 for all of the samples. The CNT weight percentages amounted to 3, 6, 9, and 12 wt.%. Although larger weight percentages of CNTs were found to be detrimental to the mechanical properties, the electrical conductivity increased with the weight percentage of CNTs, and this reached the highest values for 12 wt.% (volume resistivity of $6\times 10^{5\;}\Omega \cdot m$ as compared to $10^{10}\;\Omega \cdot m$ for reference sample). 

Rajan et al. [59] obtained electrically conductive composites by mixing graphene nanoplatelets (5, 10, or 15 wt.%) from a masterbatch (GNP in polypropylene), with a WPC masterbatch with 50/50 PP and a mix of spruce and fir wood flour, along with 3 wt.% of additives, including MAPP. The composites were prepared by melt compounding and hot pressing. The fabricated samples had either PP with 20 wt.% wood flour, PP with graphene, or PP with 20 wt.% of both flour and graphene. Although the authors used a masterbatch of GNP, the high loading of CNMs resulted in a decrease in tensile strength and only a small increase in the tensile modulus, which may potentially have been due to the lack of oxidation of the GNPs in the masterbatch. However, it was found that the highest tested loading of GNP (15 wt.%) was the percolation threshold upon which the surface resistivity decreased to 2.90 × 10^6^ Ω/sq from 1.08 × 10^14^ Ω/sq for pure a WPC sample. This level enables the use of such composites as antistatic elements. 

**Figure 4:**
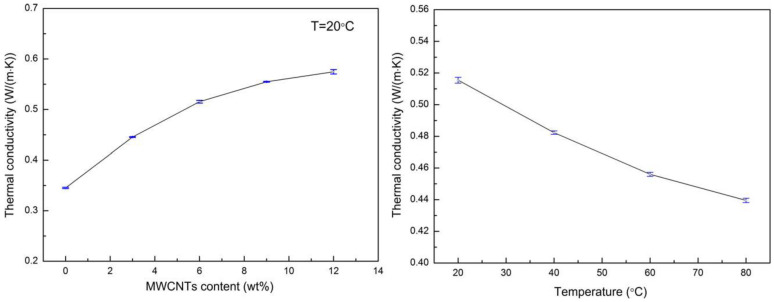
Dependence of the thermal conductivity of the WPCs on their MWCNT contents, and dependence of the thermal conductivity of the WPCs with 6 wt.% MWCNTs on temperature. Reprinted with permission from Wiley, copyright 2021 [68].

In contrast to the case of electrical transport, a simple high loading fraction of CNMs may not be enough to obtain high thermal conductivity. Agglomeration is again an issue here. Referring to Rajan et al. [59], samples with 15 wt.% graphene increased the thermal conductivity by 135%, although, as emphasised by the authors, the absolute values were still well below 1 W/mK, which is not sufficient for many applications. Analogous results were obtained by Zhang et al. [60], who manufactured composite samples from polyethylene, graphene nanoplatelets (0, 3, 6, 9, or 12 wt.%), and poplar wood fibres (40 wt.%), with maleic anhydride-grafted polyethylene (MAPE) as a compatibility agent (3 wt.%). The components were dried, mixed, and extruded using a co-rotating twin-screw extruder. The thermal conductivity increased with the wt.% of GNPs, becoming over 150% larger for 12 wt.% GNPs than for the reference sample; however, it did not increase beyond 1 W/mK. The mechanical properties for such weight percentages of GNPs showed little increase in flexural strength and modulus, or in tensile modulus. However, the tensile strength and impact strength decreased, possibly indicating an agglomeration issue. The same authors [68] fabricated samples with MWCNTs (0, 3, 6, 9, or 12 wt.%) instead of GNPs, along with an increased poplar wood fibre content (60 wt.%). In the case of these samples, the mechanical properties were all worse than for pure WPC, and again the samples showed only a small increase in thermal conductivity (Figure 4). A measurement of thermal conductivity beyond 1 W/mK (1.2 W/mK, for a sample of 40 wt.% wood particles and 15 wt.% GNP) was obtained by Al-Maqdasi et al. [53], indicating that good distribution of CNMs aids thermal transport in the samples. 

**Figure 5:**
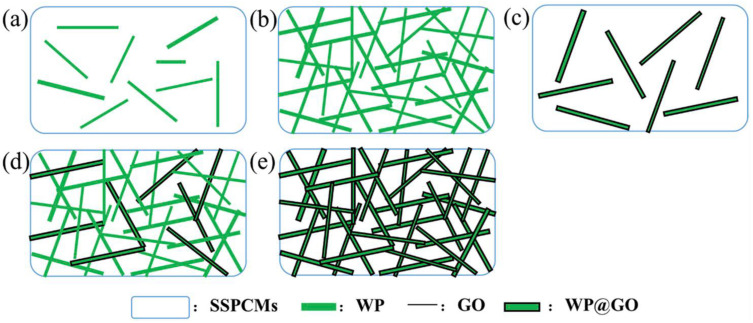
Creating a thermally conductive network by incorporating wood WP and WP@GO into the SSPCM matrix. SSPCMs: PEG-based shape-stabilised phase change materials; WP: wood powder; GO: graphene oxide; WP@GO: wood powder coated with GO. (**a**) Low loading of WPs; (**b**) high loading of WPs, forming a 3D skeleton; (**c**,**d**) although GO is coating the WPs, the thermally conductive path cannot be formed yet; (**e**) formation of a thermally conductive pathway. Reprinted with permission from Elsevier, copyright 2021 [71].

The most effective method of thermal conductivity improvement was found by Lu et al. [71]. Samples were prepared via the coating of pine wood fibres with graphene oxide, and then mixed with NCO-terminated PEG-pre polymer.and cured at 80 °C, which effectively produced GO-coated wood particles in a polyurethane matrix. It was clearly visible that the thermal conductivity of the composite increased with the weight percentage of GO, reaching the best values for 1.2 wt.% GO. However, a higher content of wood particles also contributed to an increase in the thermal conductivity, which was explained by the better percolation path formation, as shown in Figure 5. Effectively, the best result was observed for the sample with 1.2 wt.% GO, 28.8 wt.% WP, and 70 wt.% PEG-pre polymer., and this amounted to 2.3 W/mK.

A similar method for the coating of large-aspect-ratio components with CNMs was proposed by Chen et al. [58], who focused on the electrical properties and tested the possibility of producing composites for electromagnetic shielding, where the fillers were made of pure carbon fibres (CFs) and carbon fibres decorated with GO or rGO—although in the case of this study it must be taken into account that the CFs are also electrically conductive. GO and rGO were deposited on the CFs using the electrophoretic method. The cellulosic filler was made of corn straw (20 wt.% in every composite). Polyvinyl chloride was used as a polymer matrix. All components were mixed and hot-pressed in a vulcanising machine. It was found that the samples with a 4 wt.% content of CFs were the most effective in shielding; however, the use of 4 wt.% decorated CFs gave even better results. The electrical conductivity of pure CF–WPC amounted to 4.5 S/m; for GO–CF–WPC it was 6.2 S/m, while for rGO–CF–WPC it was 7.5 S/m. The rGO–CF–WPC also showed the best shielding effectiveness, with 29 dB compared to 23 dB for the CF–WPC (26% increase).

### 2.3. Photostability

**Figure 6:**
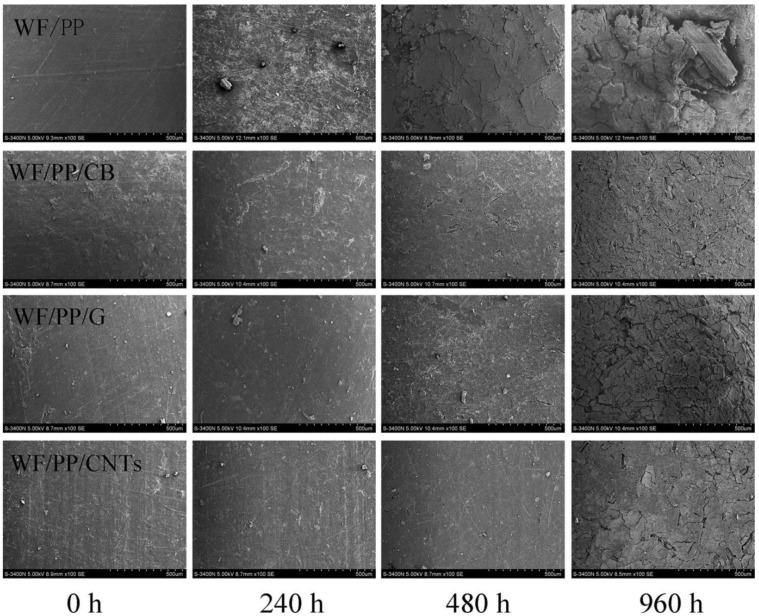
SEM images of the surface of pure WPCs (first row from the top) and below WPCs reinforced with carbon black (CB), graphite (G), and CNTs after different weathering times (WF/PP denotes wood flour/polypropylene). Reprinted by permission (Taylor & Francis Ltd., http://www.tandfonline.com) (accessed on 22 December 2021) [65].

The study of Peng et al. [65] was the only one that focused on the photostability of CNM-enriched WPCs; this work also considered the effects of various kinds of carbon nanomaterials on the performance of the hybrid composites. The reference samples for the test were composed of poplar wood flour (40 wt.%) and polypropylene (60 wt.%). Furthermore, MWCNTs, graphite, or carbon black were added to the composites (2 wt.% of the total mass). The materials were mixed and then extruded in a co-rotating twin-screw extruder, cut into pellets, and hot-pressed. The samples were then subjected to a 960 h accelerated UV weathering test. The mechanical properties test showed that the CNT-enriched samples reached the highest values of flexural and impact strength both before and after weathering. All carbon materials decreased the photo-oxidation of the surface upon weathering; however, the CNTs also showed the least surface deterioration. The scanning electron microscope (SEM) images reprinted in Figure 6 show that the surface of pure WPCs starts to become rough after 240 h, and clearly cracks after 480 h. Samples reinforced with carbon black or graphite are more resistant to weathering, and show some deterioration features after 480 h, while in the case of CNT-reinforced samples the cracks start to appear after 960 h. The CNT-enriched samples additionally had the best colour stability, although it is worth mentioning that in all cases the carbon materials made the composite surface darker. 

### 2.4. Water Absorption and Swelling Thickness

As mentioned above (publication of Kaymakci et al. [67]), the presence of MWCNTs decreases the wettability of the samples, which may be related to the strong hydrophobicity of pure MWCNTs [29,90]. Many other authors have also shown that WPCs with CNMs are characterised by lower hydrophilicity and water uptake. It is worth noting that even oxidised CNMs, which should be much more hydrophilic, decreased the water or moisture uptake of WPC samples. Sheshmani et al. [63] showed that the addition of 0.8–1 wt.% GNPs decreased the water absorption of the samples by 35%; as a result, the thickness swelling due to water uptake was diminished by 30%. Ye et al. [61] found that the absorption value of a sample containing 0.4 wt.% GO immersed in water for 24 h amounted to only 1.13 wt.%, while that of a reference amounted to 9.13 wt.%. Kushwaha et al. [77] found that the presence of plasma-treated CNTs in the bamboo-strip-based composite samples resulted in a water uptake of 23.18%, while reference samples absorbed 26.28% of water. Peng et al. [65] performed a 16-day-long water absorption test that resulted in a 2.65% water uptake for a CNT-containing sample and 3.14% for a pure WPC sample. It is worth mentioning that samples containing carbon black or graphite showed a lower water absorption than pure WPCs; however, they exhibited a higher water uptake than samples containing CNTs. Similarly CNT-enriched samples showed the least discoloration of all of the samples, both at room temperature and after a 60 °C water wash. Al-Maqdasi et al. [53] showed that the presence of GNPs in WPC samples clearly decreased the stiffness and strain drop, due to water absorption. 

### 2.5. Thermal Stability and Flammability 

Many authors also performed a thermogravimetric analysis of their samples, showing better thermal stability of the composites. Sheshmani et al. [63] found that the best stability was obtained in a sample containing 0.8 wt.% GNPs; for this sample, the degradation temperature of the polymer matrix amounted to 430 °C, while for the reference sample it was 330 °C. The authors explained that the non-agglomerated GNPs in the composite form a barrier, which delays the release and decomposition of volatile products. Ye et al. [61] found that the presence of 0.4 wt.% GO in the WPC sample increased the temperature of decomposition of both wood and polypropylene by approximately 70 °C, and they explained this phenomenon in an analogous way. Ge et al. [64]—who compared samples with graphene, carbon nanotubes, bamboo charcoal, and activated carbon—emphasised the fact that the addition of any of these carbon materials slightly decreases the decomposition temperature of the WPCs due to the fact that the carbon allotropes have a higher decomposition temperature than the raw WPC. Zhang et al. found only a slightly better thermal stability of GNP- [60] and MWCNT [68]-enriched samples, which again could be related to the agglomeration issue found for high CNM content. The same authors also found that GNP-enriched samples showed increased fire retardancy, measured by the limiting oxygen index test. Furthermore, Fu et al. tested the thermal stability and flammability of carbon-nanotube-enriched WPC [62]; the samples were prepared from sawdust of poplar (40 wt.%), 10 wt.% MAPP, and either pure CNTs or hydroxylated CNTs (CNT-OH) (0.5, 1, or 2 wt.%), as well as polypropylene. Mechanical tests showed that the tensile strength increases slightly with the addition of CNTs or CNT-OH, and reaches the highest values for 0.5 wt.% CNT or CNT-OH. However, the elongation at break decreases in all cases, which may indicate issues with agglomeration. This may be the reason for the best improvement in the thermal stability of the samples for 1 wt.% CNTs rather than for 2 wt.%, along with the quite small changes in degradation temperature. For 1 wt.% CNTs, the initial degradation temperature moved by 5 °C, while the maximum weight loss temperature changed by 10 °C. Cone calorimeter tests showed that 1 wt.% CNTs and CNT-OH could decrease the heat release rate by 16.7% and 25%, respectively, while the total heat release rate decreased by 13% and 25% for CNTs and CNT-OH, respectively. However, the presence of 1 wt.% CNTs and CNT-OH also slightly decreased the time to ignition, from 25 ± 2 s to 23 ± 2 s. 

### 2.6. Foaming Efficiency

Finally, an interesting paper by Ghalehno et al. [69] showed that graphene can also influence quite non-obvious properties of WPCs, such as their foaming efficiency at the manufacturing stage. Ghalehno et al. studied the formation of foamed WPCs formed from HDPE (50%), industrial wood flour of up to 425 μm diameter (50%), maleic anhydride-grafted polyethylene as a coupling agent (2 parts per hundred of PE), azodicarbonamide as a foaming agent (0, 1, or 3 parts per hundred of PE), and zinc oxide as a foaming agent activator (1.5 parts per hundred of azodicarbonamide). Some of the samples were also enriched with graphene nanoplatelets (1, 2, or 4 parts per hundred of PE). The samples were first melt-compounded using a twin-screw extruder, and then foamed using a compression moulding machine. The presence of graphene in the samples clearly influenced the foaming. The GNPs formed more nucleation centres for bubble growth; as a result, there was less gas available for the cells to grow, which decreased the size of the bubbles. Simultaneously, the density of the cells increased. Consequently, the volumetric density of the WPC samples clearly decreased with the addition of GNPs with the same concentration of foaming agent. The addition of GNPs also improved the tensile and flexural strength and moduli of the composite. The best improvement was obtained for 1 phr of GNPs. Larger weight fractions of the GNPs were not as effective, which was attributed to agglomeration. Agglomeration did not influence the absorption properties of the samples. In accordance with previous studies (see also Section 2.4), the water uptake and related thickness swelling decreased steadily with the amount of GNPs. 

## 3. Applications

As well as improving the performance of WPCs in current applications, we may also consider the enhancement of WPCs with new functionalities, allowing them to be used in completely new areas. For example, as indicated by Rajan et al. [59], an increase in the electrical conductivity may render the WPC’s surface antistatic and simply prevent static electricity buildup in WPC constructions, or it may enable the use of WPCs in electronic packaging. Another application in this area may be the electromagnetic shielding studied by Chen et al. [58] (see also Section 2.2). 

Moreover, WPCs filled with CNMs could potentially be used as sensors for temperature, water, or bending [91], although to the best of our knowledge they have not been tested for these applications to date. 

Another property that may be utilised in new applications is thermal conductivity. Lu et al. [71] studied the thermal properties of wood–polymer composites enriched with GO (see also Section 2.2), with a view to creating a solar thermal energy storage device. The authors designed a shape-stabilised phase change material (SSPCM) composed of polyethylene glycol (PEG)-based polyethylene glycol–polyurethane (PU), wood fibres, and graphene oxide. The PEG-based PU was used as a phase change material with a high energy storage density, with the wood fibres acting as a scaffold, while the GO enhancement increased the thermal conductivity and light absorption. As a result, a device with good thermal stability and a light–thermal conversion performance was produced.

## 4. Practical Considerations, Key Challenges, and Future Work

Currently, WPCs are widely applied, and are mostly used in indoor and outdoor furniture and flooring, frames, fences, etc. The general improvement in their properties, such as increasing their strength, or decreasing their water uptake or flammability, may definitely be of interest to the current industry. Naturally, the obstacles here may be the introduction of CNMs into industrial production and the price of the final product. However, an analysis of market reports for carbon nanotubes shows that the polymer-CNT composites currently constitute the largest share of the market [92]. This indicates that the polymer industry is already well acquainted with carbon nanomaterials and the developed procedures for their incorporation into polymers, and these could be transferred to the industrial WPC production lines. 

Our initial estimations of pricing indicate that, assuming the current market prices of CNTs, GNP, and GO, as presented in Table 2, for low weight percentages of CNMs the price of a 1 m^2^, 2 cm thick WPC would not be increased significantly. Conversely, for high weight percentages (10 wt.%), the current costs are estimated to be outside of commercial viability. However, taking into account that most studies use low filler contents, and that 1 m^2^ of WPC costs USD ~100, we can already see that the use of CNTs and graphene is quite feasible.

However, before CNM-enriched WPCs or NFPCs can enter the market, there are still some significant scientific challenges that need to be addressed. 

Section 2.1, Section 2.2, Section 2.3, Section 2.4, Section 2.5 and Section 2.6 present the results of a variety of studies, showing that enriching WPCs and NFPCs with CNMs may be beneficial to their properties. Going forward, in future studies, a much more pragmatic approach to experimental planning needs to be adopted. It would be useful to consider the vast corpus of knowledge gathered in the manufacture of pure CNM and polymer composites [93,94,95,96], and to combine this with the state-of-the-art expertise already existing in the production of high-performance WPCs and NFPCs [97,98,99]. Such analysis could potentially help with the choice of specific CNMs, wood particles, compatible polymers, and methods of manufacture, as well as avoiding such issues as agglomeration or poor adhesion of components. The performance of modelling studies could also provide valuable insight. 

It would also be useful to look at aspects such as density, which is a very basic property of every composite. To date, only Kumar et al. [92] and Rajan et al. [59] have provided data showing density changes with the content of CNMs. Kumar et al. showed that the density decreases linearly with an increase in GPC content, while Rajan et al. found the opposite trend. These discrepancies may be related to many factors, including large variations in the density of the chosen CNMs and issues related to the manufacturing of the composites, e.g., the formation of voids in the composites. Analysis of the density data could therefore be useful in the analysis of the quality of the composites, as well as in understanding the mechanisms responsible for their formation. 

Taking into account the practical applications of WPCs and NFPCs, it would be also necessary to perform more studies on the weathering of hybrid samples. Apart from the UV exposure tested by Peng et al. [65] and the full water immersion effects studied by several authors (Section 2.4), weather-related degradation agents may include heat, humidity, rain erosion, freezing, and thawing. All of these factors separately or acting collectively may impact the mechanical, physical, and chemical properties of hybrid composites and, therefore, may change their visual appearance (e.g., colour, gloss) and their surface topography (e.g., roughness, surface microstructure), as well as affecting their strength, stiffness, dimensional stability, and overall service lifetime. Other degradation factors that should be considered, and have not been taken into account thus far, include fungi and mould, as well as decay/staining by air pollutants and saltwater. 

More studies should also look into the issue of biodegradation. The only study dealing with this aspect to date, by Yaghoobi et al. [74], showed that enriching NFPCs with MWCNTs decreases the rate of biodegradation. This could be a very beneficial phenomenon; however, it could also be considered as a drawback when targeting the production of biodegradable composites [100,101]. 

Finally, it may be interesting and helpful to test the hybrid composites for acoustic properties, coatability, oil staining susceptibility, wear patterns, sand erosion, and performance under drilling. All of these properties are dependent on the specific application of the designed composites. 

## 5. Conclusions

In summary, to date, a large variety of studies on WPCs/NFPCs hybridised with carbon nanomaterials have been presented. The composites were prepared using different types and amounts of wood flour and other forms of natural fillers, such as oil palm shell powder, kenaf fibres, bamboo mats, etc. The matrices were based on both thermoplastic and thermoset polymers. The composites were prepared both with and without additives such as MAPP and MAPE compatibilizers or selected hardeners. Various types of manufacturing techniques were also used, including twin-screw extrusion, compression moulding, and laser sintering. The WPCs/NFPCs were hybridised with graphene nanoplatelets, graphene oxides, MWCNTs, and functionalised CNTs, as well as CNM masterbatches.

Inclusion of the CNMs in the WPCs and NFPCs enabled the introduction of a relative improvement in the mechanical properties of the studied composites, such as tensile strength, flexural strength, and impact strength. The hybridisation with CNMs also enabled an increase in thermal conductivity. Furthermore, the addition of CNMs rendered WPCs electrically conductive, improved their thermal stability and decreased their flammability, increased their photostability, decreased their water absorption, and improved their foaming efficiency. However, the analysis of the studies showed that the level of improvement differed significantly between the hybrid WPCs/NFPCs, and that in some cases the improvement of one parameter was detrimental to another. 

In the present review we have attempted to identify the factors that may influence the level of improvement of specific properties. These included the type, size, form, and amount of a natural filler, the presence of compatibilizers, the method of manufacture, and the type of carbon nanomaterials and their quality, as well as their amount and dispersion. In the context of carbon nanomaterials, their integration and dispersion was found to be particularly important, yet challenging. In most cases, a maximum improvement in properties was obtained at a maximum loading of CNMs, which did not cause agglomeration. The agglomeration taking place above this point was detrimental to the mechanical properties and the thermal stability. Only the electrical conductivity was found not to be sensitive to agglomeration. The distribution of CNMs was also a highly important factor for thermal conductivity. In this case, the best results were obtained for hierarchical structures where CNMs were deposited on elongated natural filler fibres.

It has been highlighted that the inclusion of CNMs may serve not only to improve the performance of WPCs/NFPCs, but also to enable the manufacture of products that may serve in new applications, such as WPC-based electromagnetic shielding or antistatic materials for electronic packaging. Furthermore, these new materials may be applicable for use in various sensors, heaters, and nanodevices. 

Section 4 included some practical considerations such as costs, listed the key challenges, and identified areas for future research. The calculations demonstrated that hybrid composites should not be expensive to produce—a fact that should facilitate their rapid commercialisation. However, for the manufacture of the hybrid composites to be successful, any manufacturing needs to be preceded by further research leading towards better control over their properties. Similarly important would be the study of a wider range of degradation agents that can affect the performance of the hybrids. 

## Figures and Tables

**Table 1:** Carbon-nanomaterial-reinforced wood–plastic composites developed in recent years.

Publication	CNM	Filler	Polymer	Other Additives	Method of Production *	Properties Investigated
Sheshmani et al. [63]	Graphene (0.2, 0.4, 0.6, 0.8, 1, 2, 4 or 5 wt.%)	Poplar flour (20 wt.%)	PP (72–77 wt.%)	MAPP (3 wt.%)	A	Mechanical properties; thermal stability
Ye et al. [61]	GO(0.1, 0.2, 0.3, and 0.4%).	Poplar powder	PP (60 wt.%)	PEI	D	Mechanical testing; electrical conductivity; water absorption
Nourbakhsh et al. [54]	MWCNT (1.5, 2.5 or 3.5 wt.%)	Poplar fibres (40 wt.%) or bagasse stalk (40 wt.%)	PP (53.5–60 wt.%)	MAPP (3 wt.%)	A	Mechanical properties
Ge et al. [64]	CNTs, graphene, activated carbon, or bamboo charcoal (2 wt.%)	Decayed particles (30, 40, or 50 wt.%)	PVC (50, 60, 70 wt.%)	Chitosan (3 wt.%)	A	Mechanical properties; thermal stability
Peng et al. [65]	MWCNTs, graphite or carbon black (2 wt.%)	Wood flour (40 wt.%)	PP (60 wt.%)	none	A	Weathering durability; mechanical properties; washing resistance
Al-Maqdasi et al. [66]	Masterbatches of GNP oxidised at the edges (7.6, 9.6 and 15 wt.%)	Sawdust of spruce and pine (25 and 40 wt.%)	HDPE (43.5–58.5 wt.%)	MAPE (1, 1.5 wt.%)	B	Mechanical properties; thermal properties
Al-Maqdasi et al. [53]	GNP Masterbatches (0,7.6,15 wt.%)	Sawdust of spruce (40 wt.%)	HDPE (43.5–58.5 wt.%)	MAPE (1.5 wt.%)	B	Mechanical properties; thermal properties
Zhang et al. [55]	MWCNT (0.1wt.%)	Pine wood powder (~15 wt.%)	(PES) (approx. 85 wt.%)	none	C	Mechanical properties
Kaymakci et al. [67]	MWCNT (0 or 1, 3, 5 wt.%)	Pine flour (50 wt.%)	PP (50 wt.%)	MAPP (3 wt.%)	A	Surface roughness; wettability; scratch resistance
Zhang et al. [57]	MWCNTs, flake graphite, or carbon black (3,6,9,12 wt.%)	Poplar fibres (52–60 wt.%)	PE (36–40 wt.%)	MAPE (3 wt.%)	A	Mechanical properties; electrical conductivity
Rajan et al. [59]	GNP (0,5,10,15 wt.%)	Spruce and fir wood flour (20 wt.%)	PP (65–80 wt.%)	MAPP (3 wt.%)	A	Electrical conductivity; mechanical properties; thermal properties
Zhang et al. [60]	GNP (0, 3, 6, 9, 12 wt.%)	Poplar wood fibres(40 wt.%)	PE (45–57 wt.%)	MAPE (3 wt.%)	B	Thermal properties
Zhang et al. [68]	MWCNTs (0, 3, 6, 9, 12 wt.%)	Poplar wood fibres (60wt.%)	PE (25–37 wt.%)	MAPE (3 wt.%)	A	Thermal properties; mechanical properties
Fu et al. [62]	Pure CNTs or CNTs-OH(0.5, 1 or 2 wt.%)	Sawdust of poplar (40 wt.%),	PP (48–50 wt.%)	MAPP (10 wt.%)	A	Flammability; thermal stability
Ghalehno et al. [69]	GNP(0, 1, 2, 4 phr)	Wood flou61r (50 wt.%)	HDPE (50 wt.%)	MAPE, ZnO (0–3 wt.%)	B	Mechanical properties
Kumar et al. [70]	GNP (0.5wt.%)	Alkalised wood powder (0, 2.5, 5, 7.5, 10 wt.%)	Epoxy resin (89.5–99.5 wt.%)	Hardener HY-951	D	Thermal, mechanical, and electrochemical properties
Zhang et al. [56]	Flake graphite (0,5,10,15,20 wt.%)	Poplar fibres (50 wt.%)	PE (27–47 wt.%)	MAPE (3 wt.%)	A	Thermal properties; mechanical properties
Lu et al. [71]	GO (0.2, 0.4, 0.8, 1.2 wt.%)	pine powder (4.8–28.8 wt.%)	PU (70–95 wt.%)	None	D	Thermal properties
Yaghoobi et al. [72]	MWCNT (0.5, 1.0, 1.5, 2.0 wt.%)	Kenaf fibre78 (30 wt.%)	PP (63–70 wt.%)	MAPP (5 wt.%)	A	Mechanical properties; thermal storage; biodegradability
Nabinejad et al. [73]	MWCNT (0.2, 0.4, 0.6, 0.8 wt.%)	Oil palm shell powder (0–15 wt.%)	Polyester resin	(MEKP) 1 wt.%	D	Mechanical properties; thermal properties
Yaghoobi et al. [74]	MWCNT (0.5, 1.0, 1.5, 2.0 wt.%)	Kenaf fibre (30 wt.%)	PP(63–65 wt.%)	MAPP (5 wt.%)	A	Mechanical properties; thermal properties
Wang et al. [75]	GO (0.05, 0.1, 0.3, 0.5, 0.7 wt.%)	Alkali-treated bamboo fibre (30 wt.%)	PP (70 wt.%)	None	D	Mechanical properties; thermal properties
Song et al. [76]	Pure CNTs or CNTs-OH(0.5, 1 or 2 wt.%)	Wood flour (40 wt.%)	PP (48–60 wt.%)	MAPP (10 wt.%)	A	Mechanical properties; wettability

* Method of production according to Figure 2. ADCA: azodicarbonamide; MAPP: maleic anhydride-grafted polypropylene; MAPE: maleic anhydride-grafted polyethylene; MEKP: methyl ethyl ketone peroxide; PEI: polyethylenimine; PP: polypropylene; PE: polyethylene; HDPE: high-density polyethylene; PU: polyurethane; PES: polyethersulfone; PU: NCO-terminated PEG-pre-based polyurethane; ZnO: zinc oxide.

**Table 2:** Estimated prices of CNM additives to WPC.

CNM	Manufacturer	Price per 1 kg (USD)	Estimated Price of CNM per 1 m^2^ of 2 cm Thick WPC (USD)	
			with 0.1 wt.% CNM	with 10 wt.% CNM
CNTs	Nanocyl SA	120	1.44	144
GNP	Cheap Tubes Inc.	500	6.00	600
GO	Graphenea	3300	39.60	3960
